# Cytotoxic Activity of Extracts from Plants of Central Argentina on Sensitive and Multidrug-Resistant Leukemia Cells: Isolation of an Active Principle from* Gaillardia megapotamica*

**DOI:** 10.1155/2018/9185935

**Published:** 2018-05-10

**Authors:** María Laura González, Mariana Belén Joray, Jerónimo Laiolo, María Inés Crespo, Sara María Palacios, Gustavo Miguel Ruiz, María Cecilia Carpinella

**Affiliations:** ^1^Fine Chemical and Natural Products Laboratory, School of Chemistry, IRNASUS-CONICET, Catholic University of Córdoba, Avda. Armada Argentina 3555, X5016DHK Córdoba, Argentina; ^2^Herbarium Marcelino Sayago, School of Agricultural Science, Catholic University of Córdoba, Avda. Armada Argentina 3555, X5016DHK Córdoba, Argentina

## Abstract

Plants are a significant reservoir of cytotoxic agents, including compounds with the ability to interfere with multidrug-resistant (MDR) cells. With the aim of finding promising candidates for chemotherapy, 91 native and naturalized plants collected from the central region of Argentina were screened for their cytotoxic effect toward sensitive and MDR P-glycoprotein (P-gp) overexpressing human leukemia cells by means of MTT assays. The ethanol extracts obtained from* Aldama tucumanensis*,* Ambrosia elatior*,* Baccharis artemisioides*,* Baccharis coridifolia*,* Dimerostemma aspilioides*,* Gaillardia megapotamica*, and* Vernonanthura nudiflora* presented outstanding antiproliferative activity at 50 *μ*g/mL, with inhibitory values from 93 to 100%, when tested on the acute lymphoblastic leukemia (ALL) cell line CCRF-CEM and the resistant derivative CEM-ADR5000, while 70–90% inhibition was observed against the chronic myelogenous leukemia (CML) cell K562 and its corresponding resistant subline, Lucena 1. Subsequent investigation showed these extracts to possess marked cytotoxicity with IC_50_ values ranging from 0.37 to 29.44 *μ*g/mL, with most of them being below 7 *μ*g/mL and with ALL cells, including the drug-resistant phenotype, being the most affected.* G. megapotamica* extract found to be one of the most effective and bioguided fractionation yielded helenalin** (1)**. The sesquiterpene lactone displayed IC_50_ values of 0.63, 0.19, 0.74, and 0.16 *μ*g/mL against K562, CCRF-CEM, Lucena 1, and CEM/ADR5000, respectively. These results support the potential of these extracts as a source of compounds for treating sensitive and multidrug-resistant leukemia cells and support compound** 1** as a lead for developing effective anticancer agents.

## 1. Introduction

Leukemia is a malignant disorder with a significant number of deaths annually [1]. According to GLOBOCAN, about 352,000 new cases of leukemia and 265,000 deaths occurred worldwide in 2012 [2].

Despite overall improvement in the outcome of conventional leukemia therapies [3, 4], some patients have poor survival rates [5] or suffer from side effects of the drugs administered [3, 4, 6], and relapse is often observed [7]. In addition, a major problem in the treatment of leukemia is the development of resistance to chemotherapeutic agents. Many cancers, including leukemia, are resistant to a wide array of chemically and functionally unrelated agents, a phenomenon known as multidrug resistance (MDR).

Various mechanisms underlie this type of resistance, the most common being overexpression of the P-glycoprotein (P-gp) transporter (ABCB1/MDR1) [8]. This protein, located in the cell membranes, is involved in the traffic of chemotherapeutic drugs outside the cancer cell [6] or in the sequestration of these in cytoplasmic organelles, preventing their therapeutic effect [9]. More than a third of cells from patients with leukemia were positive to P-gp [10]. High levels of this pump were detected in about 50% of patients with chronic myelogenous leukemia (CML) unresponsive to chemotherapy [11], while 12 and 29% of patients with acute myeloid leukemia (AML) showed high and intermediate P-gp expression, respectively [12]. For these reasons, academy and industry still concentrate on finding promising candidates for an effective and safe therapy for leukemia.

A considerable number of plants have been recognized with rich potential as a therapeutic resource, and many of their derived molecules were active on leukemia cells, including resistant phenotypes [6]. Many compounds with medicinal properties have been obtained from flora from Argentina [8, 13–16], but this resource is far from being completely explored.

The present work reports antiproliferative activity on sensitive and P-gp overexpressing leukemia cell lines of a panel of 91 extracts obtained from native and naturalized plants of Central Argentina. One of the most potent extracts,* Gaillardia megapotamica*, was submitted to bioguided fractionation to further isolate the active principle responsible for its cytotoxicity.

## 2. Materials and Methods

### 2.1. Plant Material and Extract Preparation

Plants were collected in the hills of Córdoba Province, Argentina, from November to March 2016-2017. Voucher specimens have been deposited in the “Marcelino Sayago” Herbarium of the School of Agricultural Science, Catholic University of Córdoba, and were authenticated by the botanist, G. Ruiz. Plants were selected according to their availability, accessibility, and especially the lack of scientific information about their activity and/or chemical pattern. Crushed, air-dried material (200 g) was extracted by 48 h maceration with 700 mL of 96% ethanol. The yields of each active extract, obtained after exhaustive solvent removal and expressed as percentage weight of air-dried crushed plant material, are shown in Table 2. The phytochemical profiles of the active extracts were obtained by HPLC (see supplementary material (available here)). Extract solutions were prepared immediately prior to testing.

### 2.2. Chemicals, Equipment, and Reagents

3-(4,5-Dimethyl-2-thiazolyl)-2,5-diphenyl-2H-tetrazolium bromide (MTT) and lectin from* Phaseolus vulgaris* (PHA) were purchased from Sigma Aldrich, (Sigma-Aldrich Co., St Louis, MO). Doxorubicin hydrochloride (DOX, 99.8%, Synbias Pharma Ltd.) was obtained from Nanox Release Technology (Buenos Aires, Argentina) and was used dissolved in bidistilled water. RPMI-1640 and cell culture reagents Gibco® were purchased from Invitrogen Life Technologies (Carlsbad, CA). Sterile plastic material was purchased from Greiner Bio-One (Frickenhausen, Germany). All solvents were HPLC grade. ^1^H- and ^13^C-NMR and two-dimensional spectra were recorded with a Bruker AVANCE II 400 spectrometer (Bruker Corporation, Ettlingen, Germany) with tetramethylsilane (TMS) as the internal reference. HPLC was performed on a Shimadzu LC-10 AS (Shimadzu Corp., Tokyo, Japan), equipped with a Luna C18, 250 × 4.6 mm reversed-phase column. The mobile phases were ACN/H_2_O 30 : 70, ACN/H_2_O 10 : 90, and ACN/H_2_O 70 : 30 with UV detection at 210 and 280 nm.

### 2.3. Bioguided Isolation of the Active Principle from* Gaillardia megapotamica*

**Figure 1:**
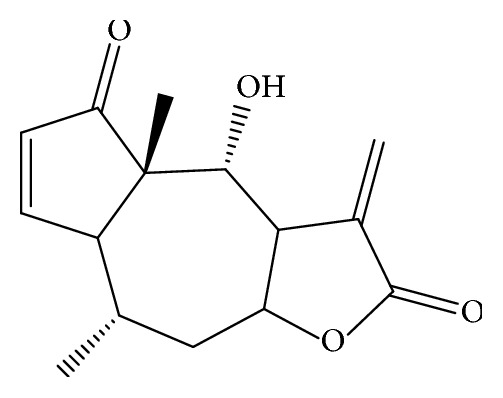
Chemical structure of helenalin** (1)**.

The cytotoxic ethanol extract of* G. megapotamica* (4 g) was initially subjected to vacuum liquid chromatography on silica gel (622 g, 63–200 *μ*m, 11.0 Å~24.0 cm; Macherey & Nagel) eluted with a step gradient of hexane/diethyl ether (Et_2_O)/methanol (MeOH) to yield 12 fractions, which were combined in 8 groups according to their thin layer chromatography (TLC) profile (F1 to F8). Fractions F1 to F5 and F7 and F8 were not active at the tested concentration (10 *μ*g/mL), while fraction F6, eluted with 100% Et_2_O, demonstrated cytotoxic effect at this concentration. Therefore, F6 was further processed by radial preparative chromatography using an isocratic mobile phase of hexane/Et_2_O 30 : 70. The fractions obtained were combined in 10 groups in accordance with the TLC analysis (F6-1 to F6-10). From fraction F6-6, a pure compound** 1** was obtained by spontaneous crystallization (Rt = 9.65, 98.2% purity, by HPLC). This compound, the remaining F6-6, and the rest of the fractions were further tested for their cytotoxic activity at 10 *μ*g/mL. Only** 1** exerted a toxic effect at this concentration. According to ^1^H and ^13^C NMR spectra (copies of the original spectra are obtainable from the corresponding author), the compound was identified as the sesquiterpene lactone helenalin C_15_H_18_O_4_ (**1**; *m*/*z* 262) [41] (yield 0.79 g/100 g of dried and crushed plant material, by HPLC) (Figure 1).

### 2.4. Cell Lines and Culture Conditions

The cytotoxicity of plant extracts was tested on acute lymphoblastic leukemia (ALL) CCRF-CEM cells [42] and on CML K562 cells [43] and their MDR P-gp overexpressing variants, CEM/ADR5000 and Lucena 1, respectively [8, 44]. Cell lines were routinely maintained in RPMI-1640 medium supplemented with 10% heat-inactivated fetal bovine serum (FBS, Natacor, Córdoba, Argentina), 2 mM L-glutamine (Invitrogen Life Technologies, CA, USA), 100 U/mL penicillin (Invitrogen Life Technologies, CA, USA), and 100 *μ*g/mL streptomycin (Invitrogen Life Technologies, CA, USA) in a 5% CO_2_ humidified atmosphere at 37°C.

As previously described, CEM/ADR5000 cells were exposed once a week to doses of DOX, gradually increasing from 1.7 to 8.6 *μ*M. The latter concentration was then used for cell maintenance [44, 45]. Lucena 1 were continuously cultured in the presence of 60 nM DOX in order to maintain P-gp overexpression [8]. Both cell lines were grown in drug-free medium 3-4 days before the experiments. Cells were subcultured twice a week and used before the 20th passage. All experiments were performed with cells in the logarithmic growth phase, with cell viabilities over 90%, determined by trypan blue staining.

### 2.5. Cell Proliferation Assay

To investigate the cytotoxic potential of the extracts, fractions, and the pure compound, the MTT colorimetric assay was performed [46]. Briefly, 5 × 10^4^ cells, suspended in 100 *μ*L of growth medium, were seeded in 96-well plates containing 100 *μ*L of medium in the presence of each tested extract, fraction, or compound previously dissolved in DMSO (final concentration 1% v/v since no adverse effects on cell growth were observed at this concentration). The extracts were evaluated at a final concentration of 50 *μ*g/mL. Following the primary screening, those extracts with promising activity on all studied cells were tested at serial dilutions ranging from 50 to 0.012 *μ*g/mL. The isolated compound was tested at 0.005–10 *μ*g/mL. After 48 or 72 h for CML or ALL, respectively, 20 *μ*L of 5 mg/mL solution of MTT in sterile PBS was added to each well and further incubated for 4 h. Then, the supernatants were removed and replaced with 100 *μ*L DMSO to solubilize the resulting purple formazan crystals produced from metabolically viable cells. Absorbance was measured with an iMark microplate reader (Bio-Rad, USA) at 595 nm. Two wells were used for each sample assayed and three independent experiments were performed.

Untreated and DMSO (1%)-treated cells were used as controls, while DOX (added to reach final concentrations of 0.003 to 40 *μ*g/mL) was used as reference. The percentage of cytotoxic activity was determined by the following formula: cytotoxicity (%) = [1 − (optical density of treated cells-optical density DMSO)/(optical density of control cells-optical density DMSO)] × 100.

Half-maximal inhibitory concentrations (IC_50_), the concentrations of the tested samples required to inhibit 50% cell proliferation, were calculated from the mean values of data from wells. Resistance factors (RF) were calculated by dividing the IC_50_ of resistant cells by the IC_50_ of the corresponding sensitive cell line [47].

### 2.6. Cytotoxicity on Peripheral Blood Mononuclear Cells (PBMC)

The cytotoxicity of the extracts on peripheral blood mononuclear cells (PBMC) was evaluated by MTT assay [46]. PBMC were collected from fresh heparinized blood and separated by density gradient centrifugation (Ficoll®) as described by Rennó et al. [48]. As the current study required samples from healthy human volunteer donors, ethical approval was provided by the Catholic University of Córdoba Research Ethics Board. Signed informed consent was obtained from donors. For the cytotoxicity assay, 1 × 10^5^ PBMC/well was incubated in duplicate in 96-well plates with PHA 10 *μ*g/mL, in the presence of increasing concentrations of the extracts (0.05–50 *μ*g/mL) or 1% DMSO for 48 h. Absorbance (Abs) and percentage of cytotoxicity were determined as described above and the IC_50_ values were calculated.

### 2.7. Statistical Analysis

The results are expressed as mean ± SE. Data were analyzed with one-way analysis of variance (ANOVA) and one-tailed unpaired *t* test using GraphPad Prism software (GraphPad Prism 5.0, GraphPad Software, Inc., CA, USA), with *p* values ≤ 0.05 as statistically significant. IC_50_s were calculated by GraphPad Prism software, responding to at least five concentrations of each extract at the 95% confidence level with upper and lower confidence limits.

## 3. Results and Discussion

With the aim of finding new agents capable of interfering with the proliferation of sensitive and MDR leukemia cells, 91 ethanol extracts obtained from different plant species of Central Argentina were primarily assayed at a fixed concentration of 50 *μ*g/mL against a panel of cells consisting of CCRF-CEM and K562 and their respective P-gp overexpressing counterparts, CEM/ADR5000 and Lucena 1. The ethanol extracts obtained from* Aldama tucumanensis, Ambrosia elatior, Baccharis artemisioides, Baccharis coridifolia, Dimerostemma aspilioides, Gaillardia megapotamica*, and* Vernonanthura nudiflora* inhibited proliferation by 93–100% in ALL cell lines while 70–90% inhibition was observed in the CML cells (Table 1). As observed, all the active species belong to Asteraceae. This could be due to more than a third of the studied plants being Asteraceae (35%) while the remaining 65% were Amaranthaceae (1%), Apiaceae (1%), Apocynaceae (1%), Aristolochiaceae (1%), Asclepiadaceae (1%), Bignoniaceae (4%), Boraginaceae (1%), Capparaceae (1%), Chenopodiaceae (1%), Dipsacaceae (1%), Euphorbiaceae (2%), Fabaceae (8%), Lamiaceae (8%), Lomariopsidaceae (1%), Loranthaceae (1%), Malvaceae (4%), Meliaceae (1%), Papaveraceae (1%), Poaceae (4%), Polygalaceae (1%), Ranunculaceae (1%), Rhamnaceae (1%), Rosaceae (2%), Rutaceae (1%), Santalaceae (1%), Schizaeaceae (1%), Solanaceae (3%), Verbenaceae (4%), and Zygophyllaceae (1%). Sesquiterpene lactones are characteristic compounds among Asteraceae [49] and many of these exhibit cytotoxic properties [50, 51]. It is interesting that* Mandevilla pentlandiana* and* Microliabum candidum* displayed a complete inhibitory effect on the proliferation of both ALL cell lines with moderate activity on CML cell lines (Table 1). Bibliographic information about the traditional uses, biological activity, and chemical constituents of the most active plants is listed in Table 2.

These active extracts were further assessed for their IC_50_ values in order to determine their level of effectiveness. As observed in Table 3, IC_50_ ranged from 0.37 to 29.44 *μ*g/mL. All extracts, except* A. tucumanensis* and* A. elatior* against CML cell lines, showed a strong cytotoxic effect with IC_50_ values lower than 20 *μ*g/mL. This value is the threshold established by the National Cancer Institute (USA, NCI) plant screening program for an extract to be considered active following an incubation period of 48 and 72 h [52]. As observed, most of them showed IC_50_ values lower than 7 *μ*g/mL. The* G. megapotamica* and* D. aspilioides* extracts were the most effective among those with less toxic effect on PBMC.

The* A. elatior *extract was highly specific, since it showed outstanding proliferation-inhibiting properties against ALL cells but not against CML cells (*p* < 0.05). The extract obtained from* D. aspilioides*, although active against all the cell lines, also induced specific cytotoxic activity, being more effective on ALL cell lines than on CML cells (*p* < 0.05).* B. artemisioides* and* B. coridifolia* extracts were highly potent, being equally active (*p* > 0.05) against almost all the assayed cell lines, with IC_50_ values from 0.37 to 5.89 *μ*g/mL, and at the same time were the most toxic against PBMC. Most of the extracts showed significantly different cytotoxic effects (*p* < 0.001–0.05) with respect to DOX, with the same or lower activity in the case of CCRF-CEM and K562, but the same or greater effectiveness in the resistant cells, CEM/ADR5000 and Lucena 1. The latter were >570-fold and 29-fold more resistant to DOX than their parental cell lines, CCRF-CEM and K562, respectively (Table 3).

It is remarkable that extracts from* A. elatior *and* D. aspilioides *displayed collateral sensitivity (CS) with CEM/ADR5000 compared to the parental cell line CCRF-CEM (degree of resistance 0.47 and 0.74, resp.) and lack of cross-resistance regarding Lucena 1. Except* B. coridifolia,* to which both P-gp overexpressing cells showed cross-resistance, the rest of the extracts showed this phenomenon only in one resistant cell. This classification is taken into consideration following Hall et al., [53] who defined RF values ≥ 2 as significant cross-resistance toward a compound, while values lower than 1 indicate a CS agent.

As mentioned,* G. megapotamica* was identified as one of the most active species. After submitting this plant to bioguided isolation, helenalin** (1)** was obtained. This sesquiterpene lactone showed outstanding IC_50_ values against K562 cells and their MDR counterpart Lucena 1 of 0.63 ± 0.06 and 0.74 ± 0.06 *μ*g/mL, respectively (2.53 ± 0.05 and 2.83 ± 0.24 *μ*M, resp.) while 0.19 ± 0.01 and 0.16 ± 0.01 *μ*g/mL were obtained against CCRF-CEM and its respective drug-resistant phenotype CEM/ADR5000 (0.74 ± 0.05 and 0.59 ± 0.05 *μ*M, resp.). In coincidence with the results obtained for the complete extract, ALL cells were more sensitive to compound** 1** than CML cells.

Although none of these most active species have been subjected to bioguided isolation to obtain their cytotoxic principles, there is some related information regarding toxicity on cancer cells of some of their constituents. Leptocarpin, present in* A. tucumanensis*, displayed cytotoxic activity against HT-29, PC-3, DU-145, MDA-MB-231, and MCF7 cells, with IC_50_ values in the range of 0.72 to 2.32 *μ*g/mL, by inducing apoptotic death [54]. The values show that this compound can be considered as active, based on the 4 *μ*g/mL cutoff established by the NCI to identify good cytotoxic compounds [55]. Eupatolide, a germacranolide sesquiterpene lactone also found in this plant, showed potent cytotoxic activity with an IC_50_ of 0.47 *μ*g/mL against H.Ep.-2 cell line [56]. The flavonoid hispidulin, present in* A. elatior*, has been tested for cytotoxic activity against Jurkat E6-1, GLC4, and COLO320 cells, showing IC_50_ ranging from 10.21 to 24.62 *μ*g/mL [57, 58]. Other constituents of this plant, dihydroparthenolide and psilostachyin, were reported as inactive when tested against KB cells [59]; the same as was found for damsinic acid tested against U937, Jurkat and Molt 4 [60], while psilostachyin C showed no effectiveness against HeLa, MCF-7, or A431 cell lines [61] but was slightly effective against BW5147 cells, with an IC_50_ value of 4.89 *μ*g/mL [62]. Chlorogenic acid showed IC_50_ values ranging from 1.8–10.7 *μ*g/mL against HepG2, Hep2, HCT116, RD, and MCF7 [63].

All the Argentinian plants of* B. coridifolia *and most of* B. artemisioides* studied by Rizzo et al. [27] were positive for the fungal macrocyclic trichothecenes roridins E and H and verrucarins A and J, which showed strong cytotoxic activity with IC_50_ of 0.0018, 0.0055,0.0012 and 0.0022 *μ*g/mL, respectively against H4TG cells [64]. When tested against Hep-2, verrucarin A showed an IC_50_ value of 0.002 *μ*g/mL [65] while verrucarins A, J and roridin E were active in the range from 0.0005 to 0.0042 *μ*g/mL against KB and BC1 cells [66]. The compound schottenol glucoside, present in* B. coridifolia*, demonstrated a toxic effect against KB cells with an IC_50_ value of 2.7 *μ*g/mL [31]. The germacranolide tomenphantin A, isolated from* D. aspilioides*, showed moderate activity against KB cells, with an IC_50_ of 3.0 *μ*g/mL [67].

Some sesquiterpene lactones, among them helenalin and mexicanin, were obtained from* G. megapotamica*. Helenalin, with a mean cytotoxic activity of 0.10 *μ*g/mL against EN2 cells [68] induced apoptosis in Jurkat T cells [69] and inhibited human telomerase activity [70], among other toxic effects in cancer cells [71]. The activity reported in this work toward the resistant cell lines makes this compound attractive as a starting point for obtaining effective cytotoxics against multidrug-resistant cells. Mexicanin showed outstanding toxic effects with an IC_50_ of 0.14 *μ*g/mL against EN2 cells [68]. Dehydroleucodine exhibited cytotoxicity against a panel of tumor cells, including eight leukemia cell lines, with IC_50_ ranging from 1.22 to 4.62 *μ*g/mL [72], and luteolin showed IC_50_ of 11.70 and 9.30 *μ*g/mL against GLC4 and COLO320 cells, respectively [57].* V. nudiflora* contains many inactive compounds, such as genkwanin, with an IC_50_ against KB and P388 cells of 8.7 and 10 *μ*g/mL, respectively [73] and germanicol, with IC_50_ 29.5 and 35.6 *μ*g/mL against HCT 116 and A549 cell lines, respectively [74], as well as the effective compound velutin (IC_50_ = 1.5 *μ*g/mL against KB cells) [73]. In addition, apigenin showed IC_50_ values from 7.29 to 16.39 *μ*g/mL against Jurkat E6-1, GLC4, and COLO320 cell lines [57, 58]. A mixture of sitosterol and stigmasterol exhibited no cytotoxic effects against P-388 and HT-29 up to a concentration of 50 *μ*g/mL [75].

## 4. Conclusions

This study demonstrated that compound** 1**, isolated from* G. megapotamica,* is a promising cytotoxic with outstanding activity against K562 and CCRF-CEM and their respective MDR counterparts, Lucena 1 and CEM/ADR5000. This identifies this sesquiterpene lactone as an important lead for obtaining therapeutics to act against resistant leukemia cells.

The outstanding antileukemia effect of* D. aspilioides* and the scant information about its components and the hypersensitivity of resistant CEM/ADR5000 cells toward its extract merit further investigation to obtain potential candidates for improving leukemia chemotherapies, especially against resistant phenotypes.

## Figures and Tables

**Table 1:** Antiproliferative activity of extracts obtained from native and naturalized plants of central Argentina.

Species	Cytotoxicity (%)			
	CCRF-CEM	CEM/ADR5000	K562	Lucena 1
*Acacia aroma* Gillies *ex* Hook. & Arn	21 ± 6	63 ± 6	0	0
*Acacia atramentaria *Benth.	23 ± 2	20 ± 3	0	0
*Acalypha communis* Müll. Arg.	12 ± 1	16 ± 2	14 ± 7	18 ± 6
*Acanthostyles buniifolius* (Hook. & Arn.) R. M. King & H. Rob.)	100	90 ± 5	52 ± 3	41 ± 4
*Achyrocline satureioides *(Lam.) DC.	74 ± 1	45 ± 21	0	8 ± 2
*Achyrocline tomentosa *Rusby	100	78 ± 5	18 ± 8	42 ± 6
*Aldama tucumanensis *(Hook. & Arn.) E. E. Schill. & Panero	100	95 ± 1	74 ± 5	70 ± 2
*Aloysia citriodora* Palau	7 ± 2	9 ± 2	0	0
*Aloysia gratissima *(Gillies & Hook.) Tronc.	67 ± 4	31 ± 5	3 ± 1	0
*Ambrosia elatior *L.	97 ± 0	95 ± 0	81 ± 0	72 ± 3
*Amphilophium carolinae* (Lindl.) L. G. Lohmann	0	10 ± 11	14 ± 8	5 ± 5
*Anemia tomentosa *(Savigny) Sw.	1 ± 2	14 ± 6	0	15 ± 2
*Araujia brachystephana *(Griseb.) Fontella & Goyder	0	25 ± 6	15 ± 1	6 ± 4
*Argemone subfusiformis *G. B. Ownbey	0	18 ± 1	2 ± 4	7 ± 4
*Aristolochia argentina *Griseb.	68 ± 0	100	69 ± 1	57 ± 11
*Artemisia annua *L.	87 ± 2	74 ± 1	40 ± 2	48 ± 3
*Artemisia verlotorum *Lamotte	88 ± 3	76 ± 3	16 ± 10	58 ± 3
*Astragalus distinens *Macloskie	0	29 ± 3	1 ± 1	12 ± 0
*Baccharis artemisioides *Hook. & Arn.	93 ± 3	100	79 ± 0	78 ± 3
*Baccharis coridifolia *DC.	100	94 ± 1	77 ± 0	75 ± 1
*Baccharis flabellata *Hook. & Arn.	100	58 ± 10	27 ± 1	10 ± 4
*Baccharis linearifolia *(Lam.) Pers.	83 ± 10	53 ± 11	0	21 ± 9
*Baccharis salicifolia *(Ruiz et Pav.) Pers.	26 ± 2	26 ± 6	8 ± 9	0
*Bidens pilosa *L.	5 ± 7	0	23 ± 6	0
*Cantinoa mutabilis* (Rich.) Harley & J. F. B. Pastore	85 ± 6	99 ± 9	21 ± 8	23 ± 2
*Capparis amisquea *Kuntze	0	9 ± 2	18 ± 4	6 ± 1
*Chromolaena hookeriana* (Griseb.) R. M. King & H. Rob.	14 ± 13	10 ± 10	0	0
*Condalia microphylla *Cav.	19 ± 3	0	0	0
*Cortaderia speciosa* (Nees & Meyen) Stapf	11 ± 12	3 ± 3	0	6 ± 1
*Cotoneaster glaucophyllus* Franch.	0	0	19 ± 1	10 ± 3
*Croton lachnostachyus* Baill.	16 ± 8	39 ± 3	36 ± 3	40 ± 4
*Cynoglossum amabile* Stapf & J. R. Drumm.	0	22 ± 2	16 ± 2	9 ± 1
*Dalea elegans *Hook. & Arn.	47 ± 8	20 ± 3	3 ± 5	20 ± 6
*Dimerostemma aspilioides *(Griseb.) M. D. Moraes	96 ± 0	97 ± 1	90 ± 1	85 ± 1
*Dipsacus fullonum *L.	0	7 ± 2	18 ± 2	14 ± 2
*Dolichandra cynanchoides *Cham.	0	11 ± 1	0	10 ± 8
*Dolichandra unguis-cati *(L.) L. G. Lohmann	8 ± 9	16 ± 4	7 ± 4	5 ± 4
*Dysphania ambrosioides *(L.) Mosyakin & Clemants	12 ± 12	14 ± 11	0	0
*Elaphoglosum lorentzii *(Hieron.) H. Christ	40 ± 10	8 ± 3	0	0
*Eryngium horridum *Malme	0	18 ± 3	10 ± 2	0
*Flourensia campestris *Griseb.	83 ± 3	71 ± 4	18 ± 6	13 ± 5
*Flourensia oolepis *S. F. Blake	100	80 ± 3	15 ± 2	8 ± 5
*Gaillardia megapotamica *(Spreng.) Baker	100	100	80 ± 2	78 ± 3
*Gomphrena pulchella *Mart.	0	0	0	0
*Grindelia pulchella *Dunal	77 ± 12	91 ± 4	72 ± 2	82 ± 1
*Jarava ichu *Ruiz et Pav.	39 ± 2	11 ± 11	3 ± 3	0
*Jodina rhombifolia *(Hook. & Arn.) Reissek	33 ± 2	30 ± 6	8 ± 3	17 ± 1
*Kageneckia lanceolata* Ruiz & Pav.	68 ± 11	33 ± 2	6 ± 3	16 ± 3
*Lantana grisebachii* Seckt	96 ± 18	52 ± 12	27 ± 10	68 ± 1
*Lepechinia floribunda* (Benth.) Epling	100	84 ± 4	1 ± 5	1 ± 1
*Lepechinia meyenii *(Walp.) Epling	95 ± 0	92 ± 2	38 ± 13	31 ± 30
*Lessingianthus mollissimus *(Hook. & Arn.) H. Rob.	6 ± 2	25 ± 2	4 ± 5	0
*Ligaria cuneifolia *(Ruiz & Pav.) Tiegh.	15 ± 4	38 ± 2	13 ± 2	0
*Lippia turbinata *Griseb.	0	27 ± 18	15 ± 3	5 ± 7
*Lithrea molleoides *(Vell.) Engl.	100	52 ± 14	0	0
*Lorentzianthus viscidus *(Hook. & Arn.) R. M. King & H. Rob.	67 ± 15	18 ± 7	0	0
*Mandevilla pentlandiana *(A. DC.) Woodson	100	100	71 ± 4	60 ± 5
*Marrubium vulgare *L.	29 ± 9	40 ± 10	16 ± 7	11 ± 0
*Melia azedarach *L.	84 ± 1	11 ± 2	38 ± 12	0
*Melinis repens *(Willd.) Zizka	9 ± 7	14 ± 3	0	2 ± 2
*Melissa officinalis *L.	0	19 ± 29	0	0
*Microliabum candidum *(Griseb.) H. Rob.	100	100	72 ± 12	63 ± 14
*Minthostachys verticillata *(Griseb.) Epling	16 ± 8	55 ± 2	12 ± 11	6 ± 2
*Monnina dictyocarpa *Griseb.	30 ± 21	46 ± 14	8 ± 10	0
*Ophryosporus charua *(Griseb.) Hieron.	47 ± 14	33 ± 3	23 ± 4	36 ± 3
*Pascalia glauca *Ortega	27 ± 15	35 ± 3	12 ± 1	23 ± 1
*Pavonia aurigloba *Krapov. & Cristóbal	5 ± 3	15 ± 3	5 ± 1	0
*Podranea ricasoliana *(Tanfani) Sprague	40 ± 10	6 ± 6	0	26 ± 3
*Porlieria microphylla *(Baill.) Descole, O'Donell & Lourteig	6 ± 8	0	6 ± 8	0
*Prosopis alba *Griseb.	0	0	0	0
*Prosopis sp.*	4 ± 10	0	0	0
*Pterocaulon alopecuroides *(Lam.) DC	87 ± 3	87 ± 2	0	4 ± 2
*Pyrostegia venusta *(Ker Gawl.) Miers	10 ± 3	11 ± 1	21 ± 1	0
*Ruprechtia apetala* Wedd.	22 ± 7	15 ± 15	14 ± 7	1 ± 4
*Salvia cuspidata *Ruiz & Pav.	100	90 ± 8	14 ± 4	64 ± 0
*Schizachyrium condensatum *(Kunth) Nees	33 ± 3	34 ± 3	0	0
*Senecio madagascariensis *Poir.	14 ± 14	23 ± 4	22 ± 2	3 ± 6
*Senecio viravira *Hieron.	44 ± 26	47 ± 2	6 ± 3	0
*Senna aphylla *(Cav.) H. S. Irwin et Barneby	1 ± 2	22 ± 1	7 ± 3	10 ± 3
*Sida rhombifolia *L.	21 ± 5	1 ± 1	39 ± 4	0
*Solanum argentinum *Bitter & Lillo	53 ± 15	0	14 ± 14	17 ± 1
*Solanum palinacanthum *Dunal	76 ± 13	12 ± 1	64 ± 3	0
*Solanum sisymbriifolium *Lam.	81 ± 16	0	48 ± 1	0
*Sphaeralcea cordobensis *Krapov.	34 ± 11	1 ± 1	0	0
*Sphaeralcea cordobensis *sp.* mutant *Krapov.	21 ± 12	4 ± 4	7 ± 2	0
*Tagetes minuta* L.	26 ± 4	0	0	0
*Thalictrum decipiens *Boivin	13 ± 8	3 ± 3	0	9 ± 4
*Thelesperma megapotamicum *(Spreng.) Kuntze	37 ± 9	58 ± 12	0	5 ± 2
*Trichocline reptans *(Wedd.) Hieron.	10 ± 3	0	0	3 ± 0
*Vernonanthura nudiflora *(Less.) H. Rob.	100	96 ± 1	89 ± 4	78 ± 1
*Wedelia buphtalmiflora* Lorentz	5 ± 5	7 ± 5	2 ± 2	24 ± 2
*Zanthoxylum coco *Hook. f. & Arn.	11 ± 4	11 ± 9	87 ± 1	0

Extracts were tested at 50 *μ*g/mL. Values are expressed as mean ± SE.

**Table 2:** Native plants from Central Argentina showing antiproliferative activity.

Plant species	Yield (%); voucher: UCCOR number	Common name	Traditional uses/*in vitro* reported activities	Reported compounds^a^
*Aldama tucumanensis * (Asteraceae)	13.6; 186	-	Antirheumatic [17]/NR	Clerod-14-ene-3*α*,4*β*,13*ε*-triol; annuolide A; 17,18-dihydroleptocarpin; acetyl-17,18-dihydroleptocarpin; tomentosin; dihydroniveusin B; roseostachenone; leptocarpin; eupatolide; dihydroniveusin A; niveusin A; oplopanone; scoparone; 12*β*-ethoxy-ent-kaur-9(ll),16-dien-19-oic acid [18]. Kaurenic acid [19].

*Ambrosia elatior* (Asteraceae)	2.2; 215	altamisa	Contraceptive [20]. Headache; expectorant [21]/Antiprotozoal [22].	Cumanin [22]. 4ß,10*α*-alloaromadendrane; psilostachyin; psilostachyin C; psilostachyin B; dihydroparthenolide; hispidulin [23]. Isochlorogenic acid; chlorogenic acid [24]. 6*α-*Hydroxyeudesm-4(15)-ene-9*β*-*O*-anisate, 1*β*-hidroxy eudesma-4,11(13)-dien-12-oic acid; damsinic acid; 1*β*,6*α*-dihydroxyeudesm- 4(15)-ene [25].

*Baccharis artemisioides* (Asteraceae)	2.8; 142	Romerillo blanco	NR/Inhibition of germination [26].	Verrucarin A; verrucarin J; roridin A; roridin D; roridin E [27]. Bartemidiolide [28]. Deoxybartemidiolide [29].

*Baccharis coridifolia* (Asteraceae)	2.2; 147	Mio-mio	Pesticide [21]/Germination inhibition [26]. Antifeedant [30].	Verrucarin A; verrucarin J; roridin A; roridin D; roridin E; roridin H [27]. Schottenol glucoside [31].

*Dimerostemma aspilioides* (Asteraceae)	5.5; 246		NR/Inhibition of germination [26].	Tomenphantin A [32].

*Gaillardia megapotamica *var. *radiata* (Asteraceae)	13.76; 127	topasaire	Antialopecic, dandruff, seborrhoea [21, 33]. Antineuralgic, against headache [21]/Antifungal [34]. Ant foraging inhibitor [35].	Helenalin; nepetin; luteolin; mexicanin; 2ß-hydroxy-2,3-dihydrohelenalin [36, 37]. Dehydroleucodine [38].

*Vernonanthura nudiflora* (Asteraceae)	7.2; 129	-	NR/Antifeedant [30]	Vernudiflorid [39]. Glaucolide A; glaucolide B; lupeol; *β*-amyrin; *α*-amyrin; germanicol; sitosterol; stigmasterol; genkwanin; velutin; apigenin; (4R*∗*, 8S*∗*, lOR*∗*)-l-oxo-4-hydrox-8-tiglyloxy-13-acetoxygermacra-5E,7(13)-dien-6,12-olide; (4R*∗*, 8S*∗*, lOR*∗*)-1,4-epoxy-8-tiglyloxy-l0-hydroxy-l3-acetoxygermacra-1,5E,7(13)-trien-6,12-olide; (lR*∗*,4R*∗*. 5R*∗*, 8S*∗*, lOR*∗*)-1,4-epoxy-l-ethoxy-5-hydroxy-8- methacryloxy-13-acetoxygermacra-SE, 7(11)-dien-6,12-olide; (1S*∗*, 4R*∗*, 5S*∗*, 6S*∗*, 8S*∗*, 10R*∗*)-1,4-dihydroxy-5,10,13-triacetoxy 8-tiglyloxy-cadin-7(11)-en-6,12-olide; 8-tiglyloxycadin-7(11)-en-6,12-olide; (1S*∗*, 4R*∗*, 8S*∗*, 10R*∗*)-1,4-epoxy 8,10-diacetoxy-l-formyloxy-13-hydroxygermacra-5E,7(11)-dien-6,12-olide [40].

NR: not reported. ^a^The compounds described are those reported in organic extracts.

**Table 3:** Half-maximal inhibitory concentrations of most effective extracts.

Species	IC_50_ (*μ*g/mL) (mean ± SE)						
	CCRF-CEM	CEM/ADR5000	RF	K562	Lucena 1	RF	PBMC
*Aldama tucumanensis*	5.39 ± 0.23^a,*∗∗∗*^	17.56 ± 0.50^a,b,*∗∗∗*^	3.26	20.24 ± 1.26^a,b,*∗∗∗*^	29.44 ± 5.82^b,†^	1.45	9.13 ± 1.84
*Ambrosia elatior*	5.84 ± 1.07^a,*∗∗*^	2.73 ± 0.63^a,*∗∗∗*^	0.47	19.16 ± 2.48^b,*∗∗*,†^	21.93 ± 2.91^b,††^	1.14	4.01 ± 0.67
*Baccharis artemisioides*	1.15 ± 0.10^a,*∗∗∗*^	0.94 ± 0.24^a,*∗∗∗*^	0.82	1.90 ± 0.33^a,*∗*^	5.74 ± 0.14^b,*∗∗∗*,†††^	3.02	0.95 ± 0.21
*Baccharis coridifolia*	0.37 ± 0.03^a,*∗∗∗*^	5.89 ± 0.87^b,*∗∗∗*,†††^	15.92	0.51 ± 0.07^a^	1.08 ± 0.11^a,*∗∗∗*^	2.12	0.08 ± 0.01
*Dimerostemma aspilioides*	1.60 ± 0.07^a,*∗∗∗*^	1.18 ± 0.30^a,*∗∗∗*^	0.74	6.89 ± 0.50^b,*∗∗∗*,†^	10.38 ± 1.16^b,*∗∗*,††^	1.51	2.69 ± 0.53
*Gaillardia megapotamica*	0.70 ± 0.03^a,*∗∗∗*^	1.21 ± 0.005^b,*∗∗∗*^	1.73	2.77 ± 0.02^c,*∗∗∗*^	5.95 ± 0.17^d,*∗∗∗*,†^	2.15	2.76 ± 0.52
*Vernonanthura nudiflora*	2.21 ± 0.08^a,*∗∗∗*,††^	6.38 ± 0.20^b,*∗∗∗*,†^	2.89	6.41 ± 0.91^b,*∗∗*,†^	10.76 ± 0.78^c,*∗∗*^	1.68	13.70 ± 1.06
doxorubicin	0.07 ± 0.0006^a^	>40^†††^	>571	0.78 ± 0.14^a^	22.99 ± 1.18^b,†††^	29.47	0.39 ± 0.02

Results represent the mean ± SE. Means followed by the same letter in each row were not significantly different (Tukey, *p* > 0.05). Statistical comparisons between extracts and doxorubicin were analyzed by one-tailed unpaired *t* test. *∗∗∗* indicates *p* < 0.001, *∗∗* indicates *p* < 0.01, and *∗* indicates *p* < 0.05. Statistical comparisons between leukemia cells with respect to PBMC were analyzed by ANOVA-Tukey's multiple comparison test. ††† indicates *p* < 0.001, †† indicates *p* < 0.01, and † indicates *p* < 0.05.

## Abbreviations

ALL: Acute lymphoblastic leukemia
CML: Chronic myelogenous leukemia
CS: Collateral sensitivity
IC_50_: Half-maximal inhibitory concentration
MDR: Multidrug resistance
MTT: 3-(4,5-Dimethyl-2-thiazolyl)-2,5-diphenyl-2H-tetrazolium bromide
PBMC: Peripheral blood mononuclear cells
P-gp: P-glycoprotein
RF: Resistance factor.

## Supplementary Materials

Supplementary Materials High performance liquid chromatography (HPLC) fingerprints with UV detection at 210 and 280 nm and UV spectra of* Aldama tucumanensis, Ambrosia elatior, Baccharis artemisioides, Baccharis coridifolia, Dimerostemma aspilioides, Gaillardia megapotamica*, and* Vernonanthura nudiflora* extracts are shown. Analytical HPLC profile of the isolated compound, helenalin, with UV detection at 210 nm is also shown.
