# Supplementary material for: Cytotoxic Activity of Extracts from Plants of Central Argentina on Sensitive and Multidrug-Resistant Leukemia Cells: Isolation of an Active Principle from Gaillardia megapotamica
Source: Evid Based Complement Alternat Med. 2018 May 10;2018:9185935. doi: 10.1155/2018/9185935 (PMC5971282; doi:10.1155/2018/9185935)
Supplement: Supplementary Materials — High performance liquid chromatography (HPLC) fingerprints with UV detection at 210 and 280 nm and UV spectra of Aldama tucumanensis, Ambrosia elatior, Baccharis artemisioides, Baccharis coridifolia, Dimerostemma aspilioides, Gaillardia megapotamica, and Vernonanthura nudiflora extracts are shown. Analytical HPLC profile of the isolated compound, helenalin, with UV detection at 210 nm is also shown. [file 9185935.f1.docx]

Cytotoxic Activity of Extracts from Plants of Central Argentina on Sensitive and Multidrug-Resistant Leukemia Cells: Isolation of an Active Principle from *Gaillardia megapotamica*

María Laura González,^1^ Mariana Belén Joray,^1^ Jerónimo Laiolo,^1^ María Inés Crespo,^1^ Sara María Palacios,^1^ Gustavo Miguel Ruiz,^2^ and María Cecilia Carpinella^1,*^

^1^ *Fine Chemical and Natural Products Laboratory, School of Chemistry, IRNASUS-CONICET. Catholic University of Córdoba, Avda. Armada Argentina 3555, X5016DHK Córdoba, Argentina*

*^2^ Herbarium Marcelino Sayago, School of Agricultural Science, Catholic University of Córdoba, Avda. Armada Argentina 3555, X5016DHK Córdoba, Argentina*

^*^Corresponding author. María Cecilia Carpinella. Laboratorio de Química Fina y Productos Naturales, Facultad de Ciencias Químicas, Universidad Católica de Córdoba, Avda. Armada Argentina 3555, X5016DHK, Córdoba, Argentina. Tel: 54 351- 4938000. Ext 611. Fax: +54 3514938061. E-mail address: [ceciliacarpinella@ucc.edu.ar](mailto:ceciliacarpinella@campus1.uccor.edu.ar).


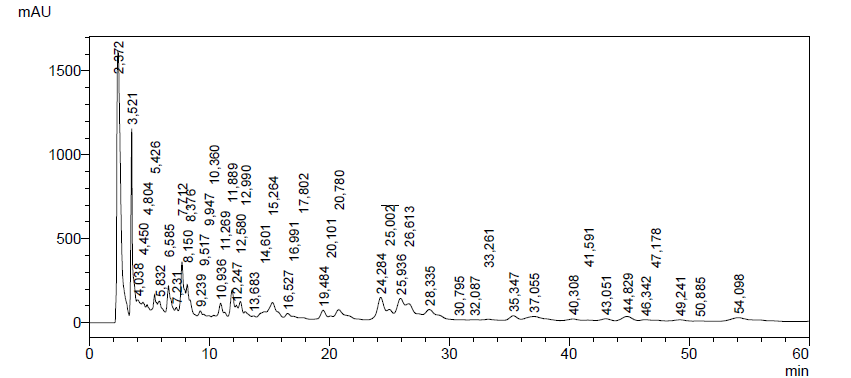
A


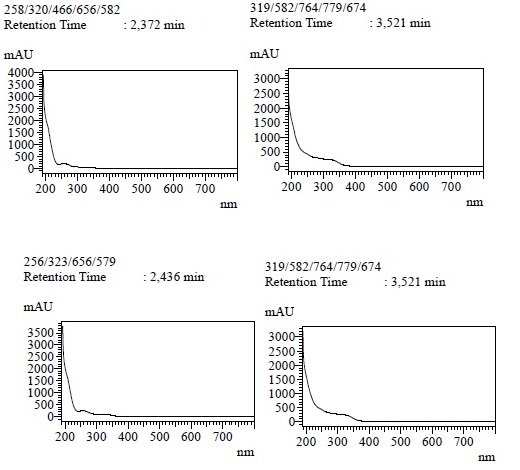


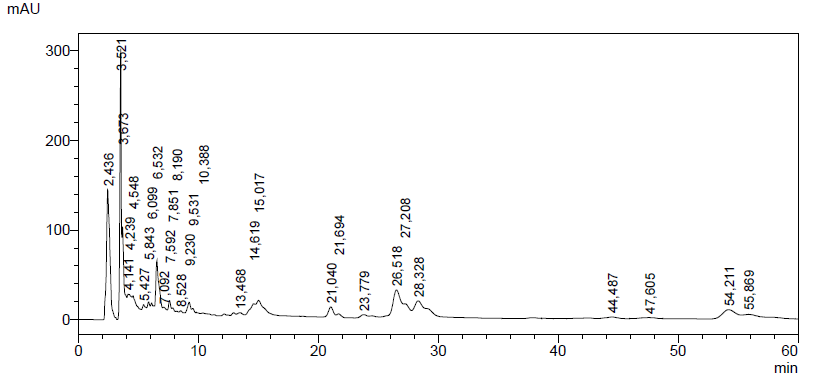
B


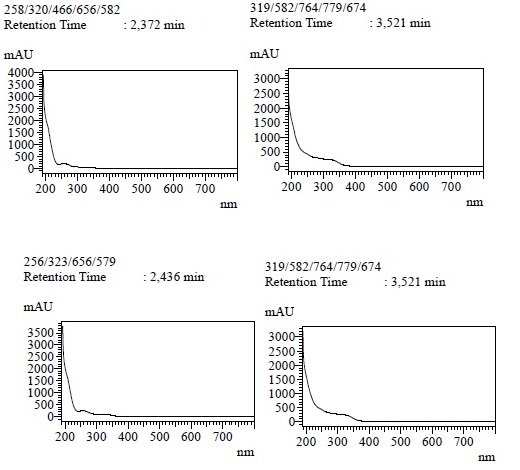

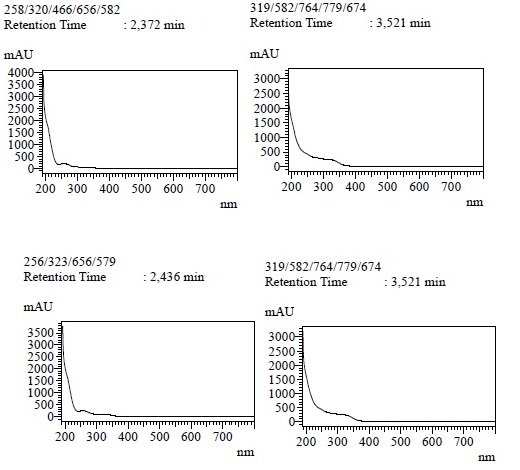


Analytical HPLC profile of *Aldama tucumanensis* extract at 20 mg/mL in methanol. Mobile phase ACN/H_2_O 30:70. UV detection at 210 (A) and 280 (B) nm.


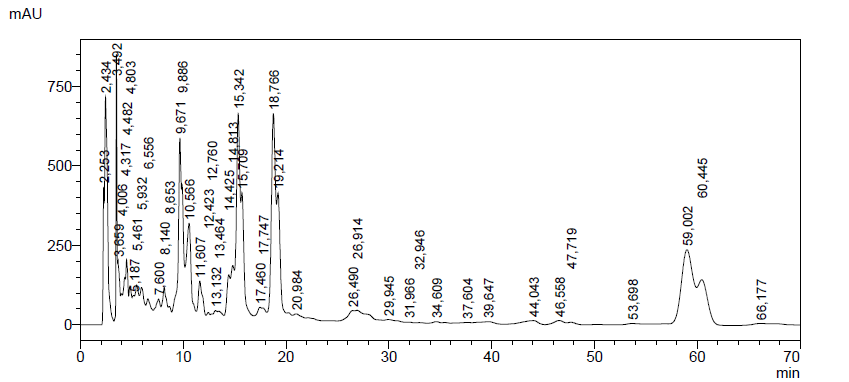
A


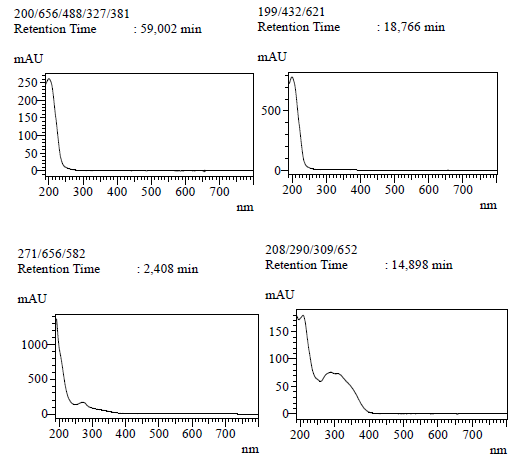

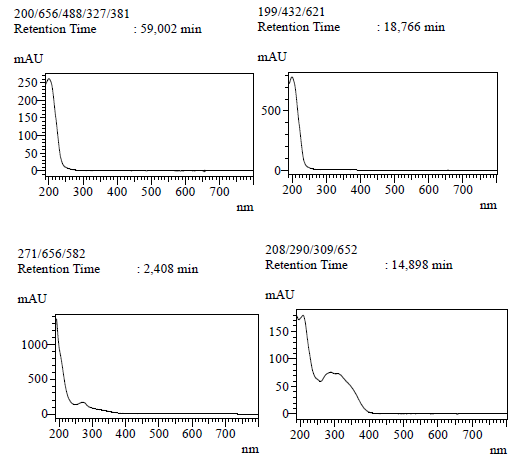


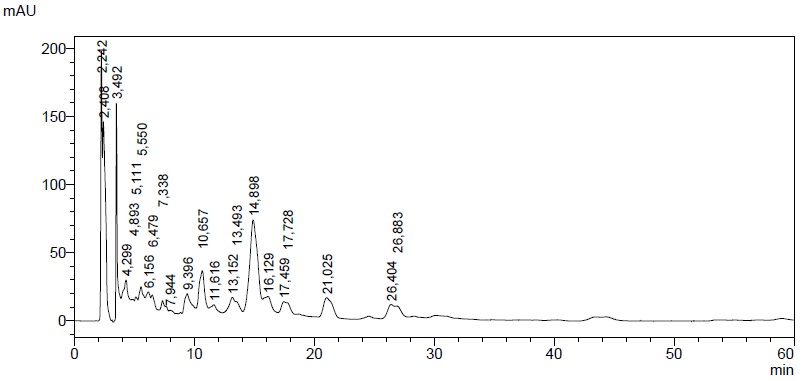
B


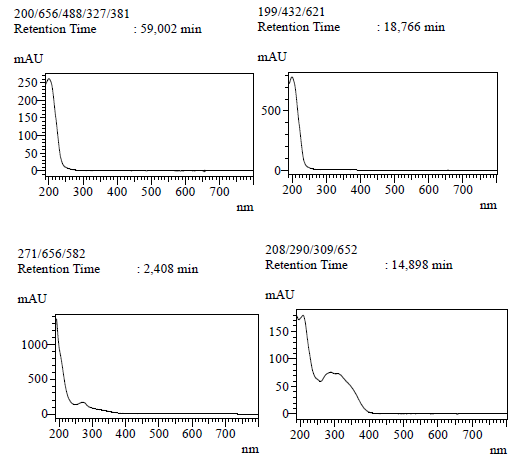

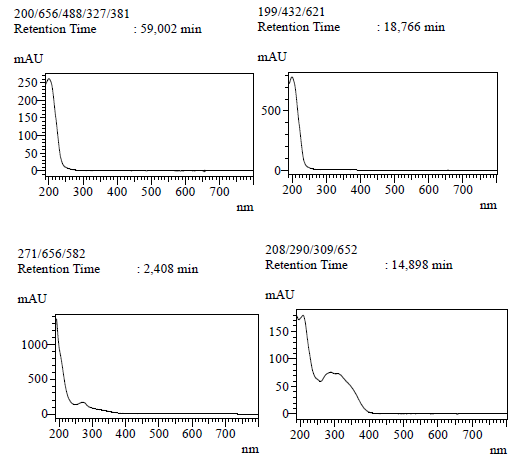


Analytical HPLC profile of *Ambrosia elatior* extract at 10 mg/mL in methanol. Mobile phase ACN/H_2_O 30:70. UV detection at 210 (A) and 280 (B) nm.


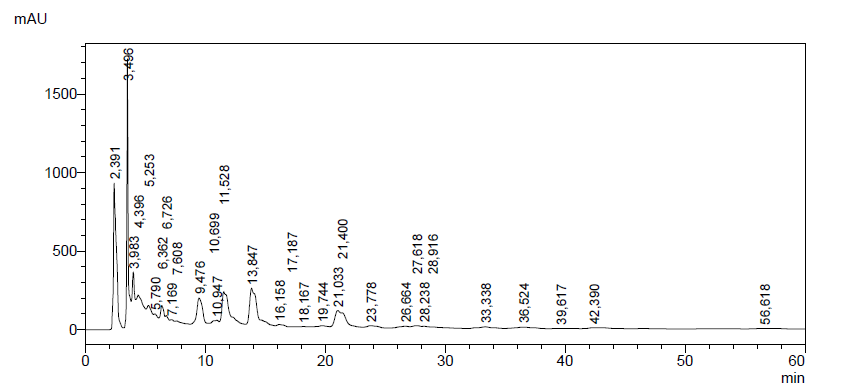
A


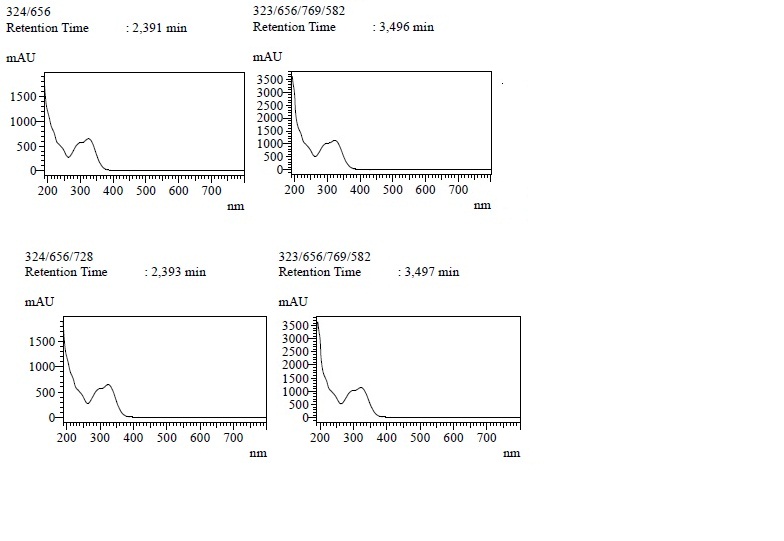


B


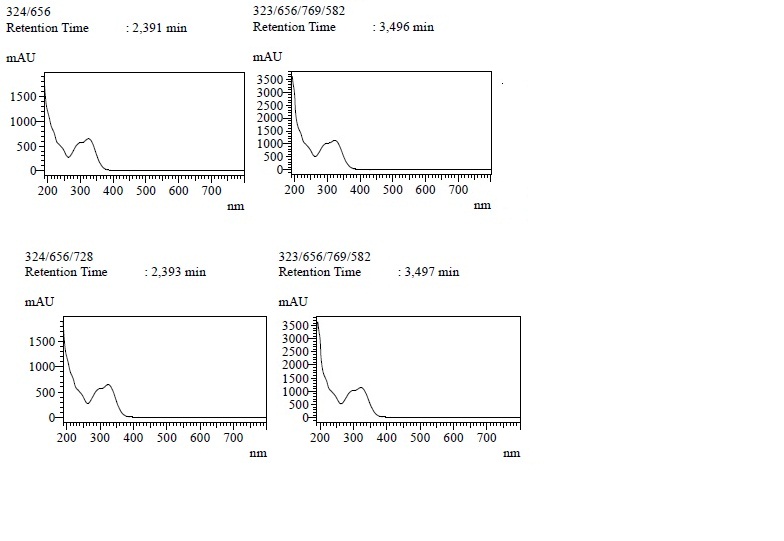

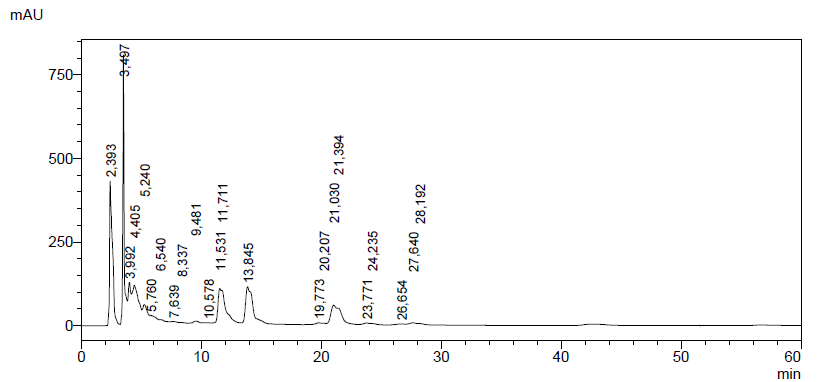


Analytical HPLC profile of *Baccharis artemisioides* extract at 10 mg/mL in methanol. Mobile phase ACN/H_2_O 30:70. UV detection at 210 (A) and 280 (B) nm.


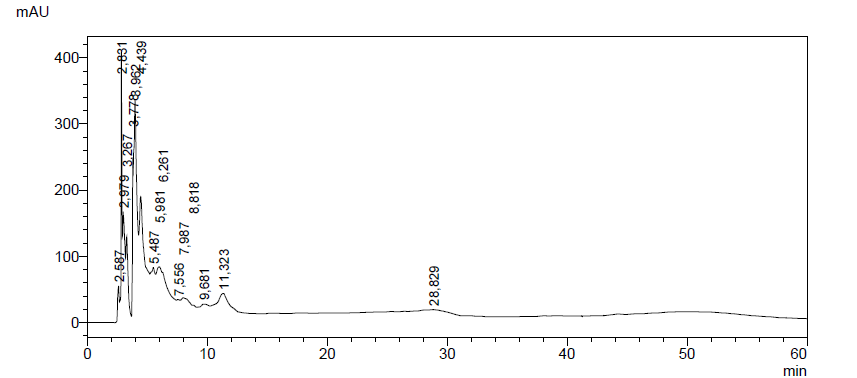
A


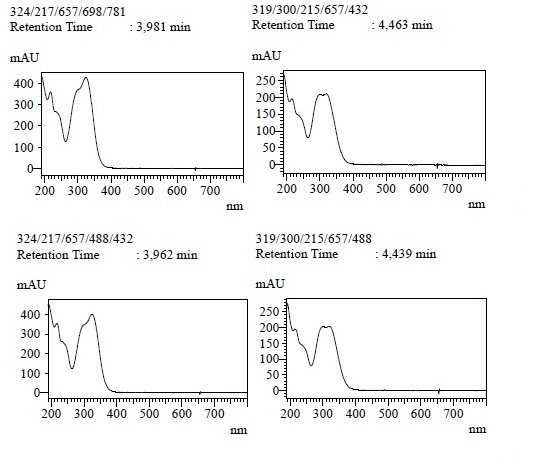


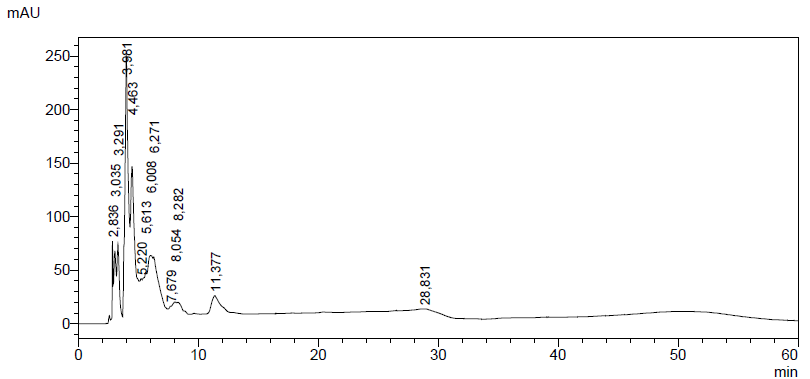
B


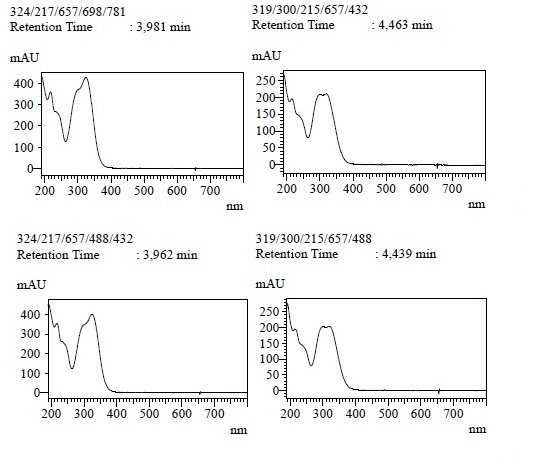


Analytical HPLC profile of *Baccharis coridifolia* extract at 10 mg/mL in methanol. Mobile phase ACN/H_2_O 10:90. UV detection at 210 (A) and 280 (B) nm.


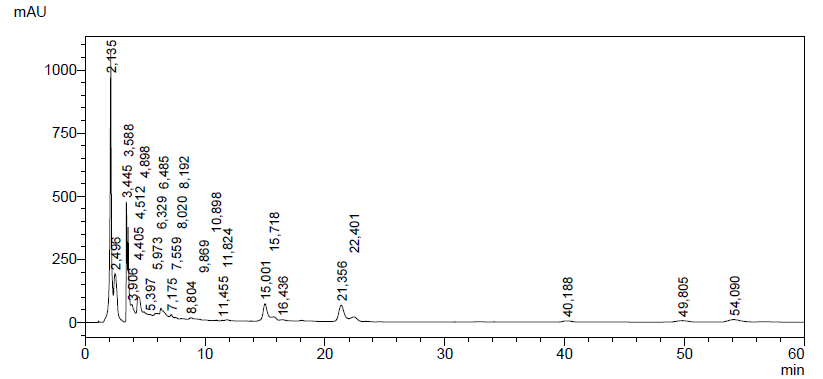
A


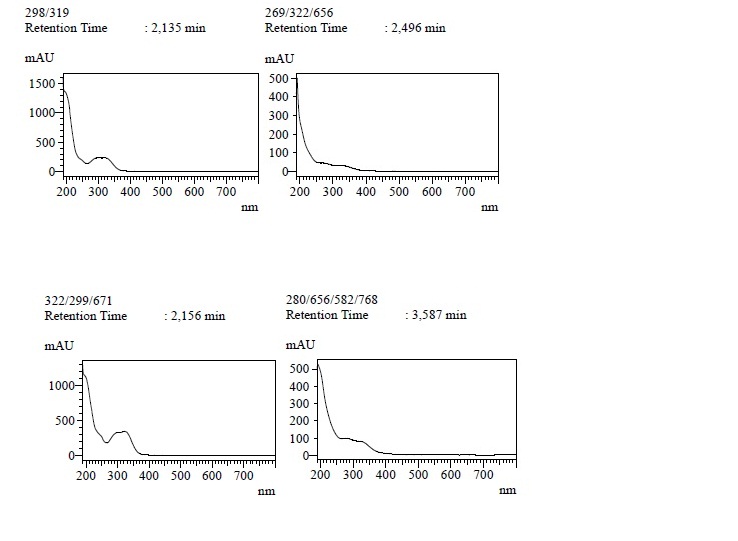


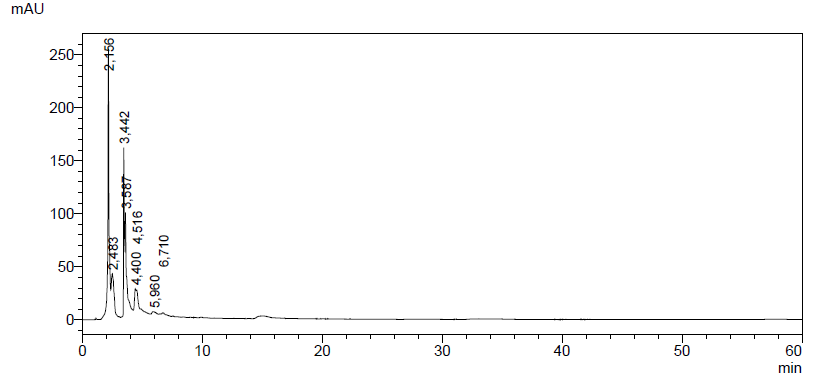
B


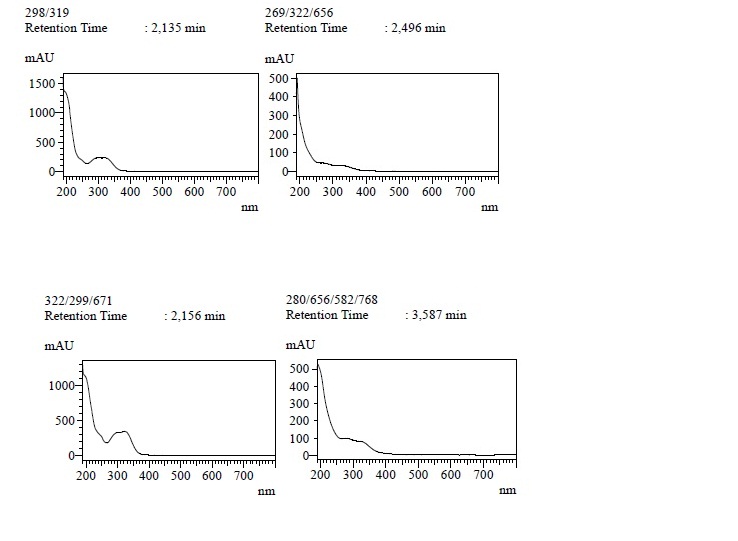


Analytical HPLC profile of *Dimerostemma aspilioides* extract at 2.5 mg/mL in methanol. Mobile phase ACN/H_2_O 70:30. UV detection at 210 (A) and 280 (B) nm.


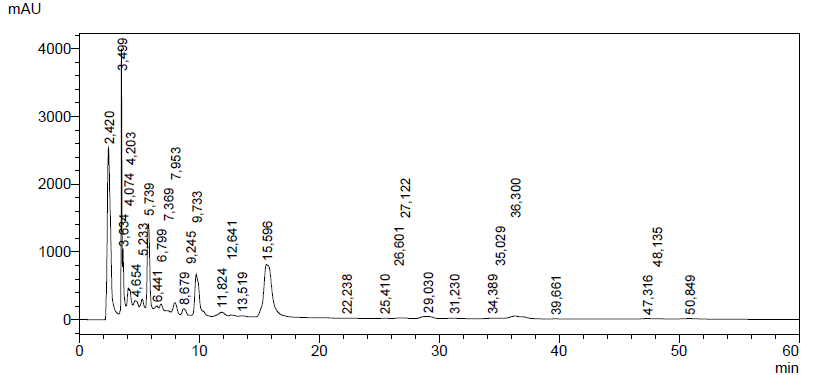
A


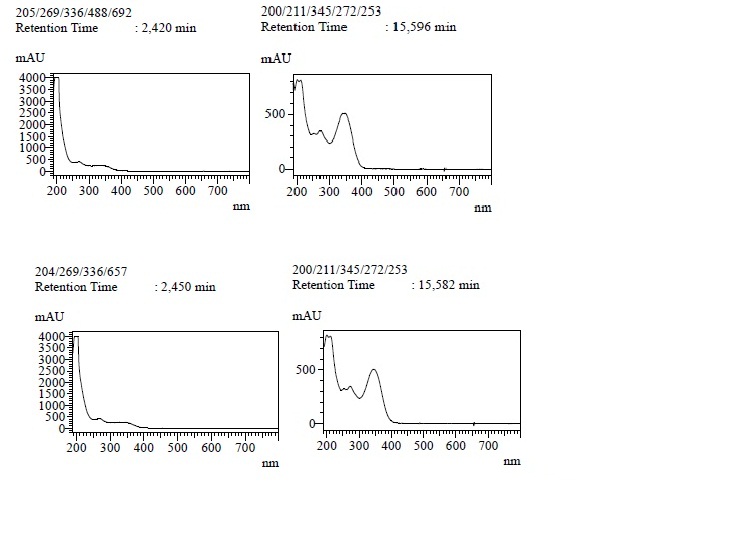


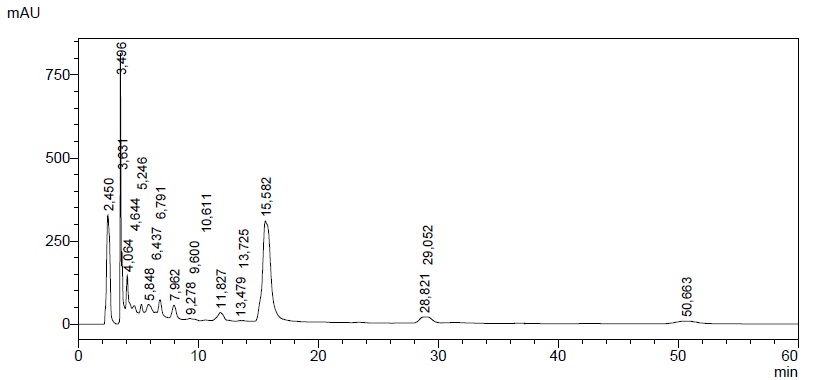
B


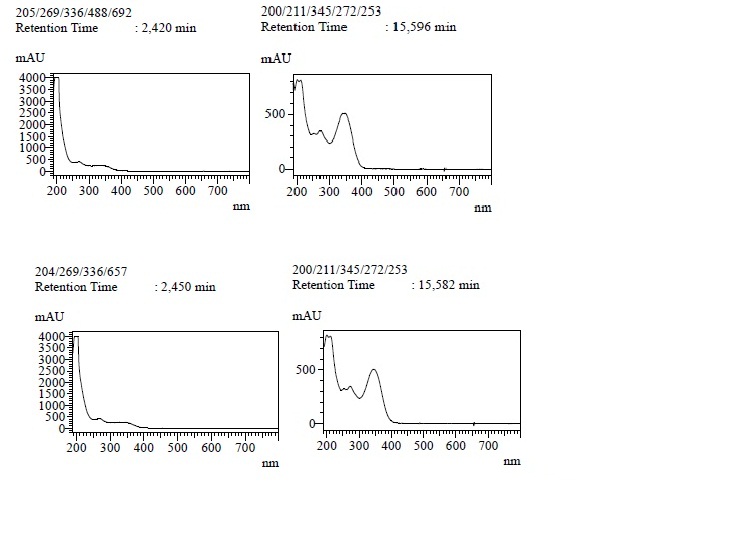


Analytical HPLC profile of *Gaillardia megapotamica* extract at 10 mg/mL in methanol. Mobile phase ACN/H_2_O 30:70. UV detection at 210 (A) and 280 (B) nm.


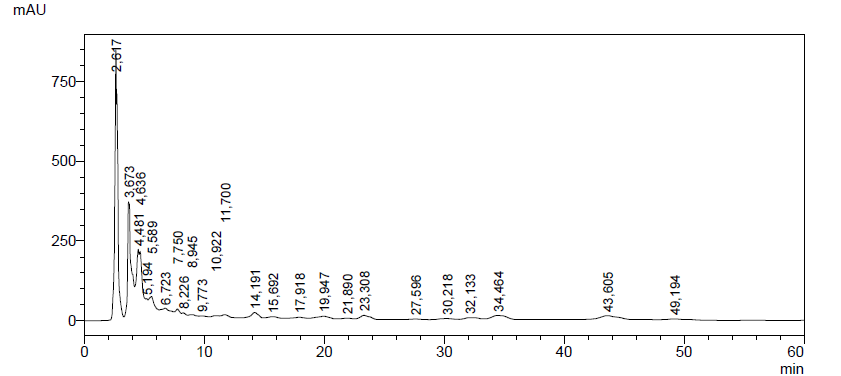
A


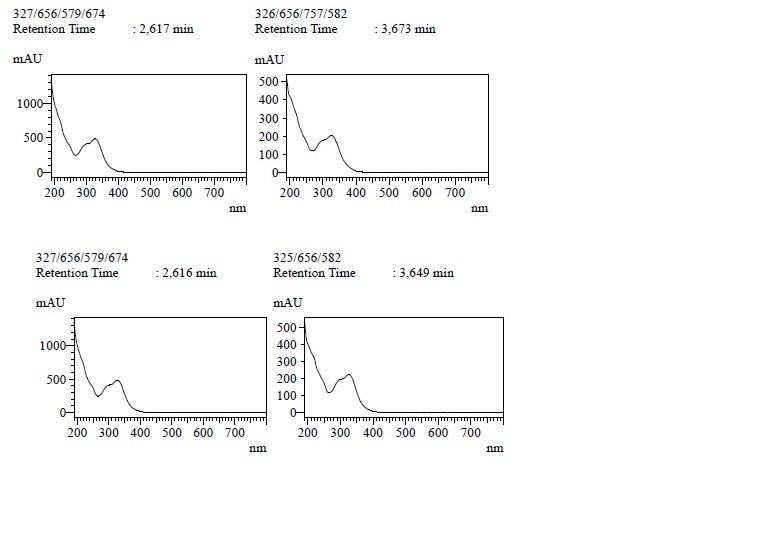


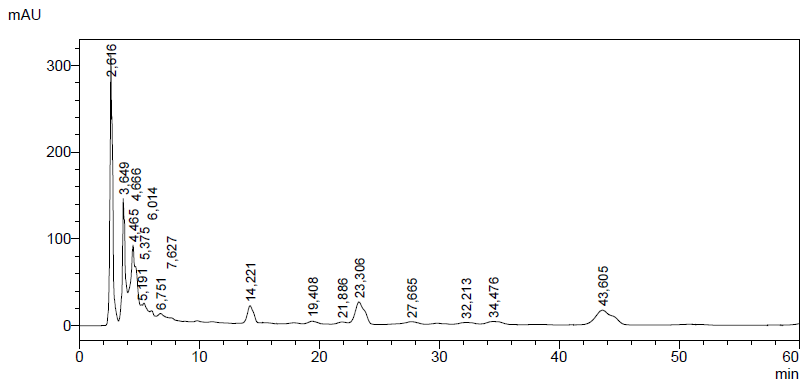
B


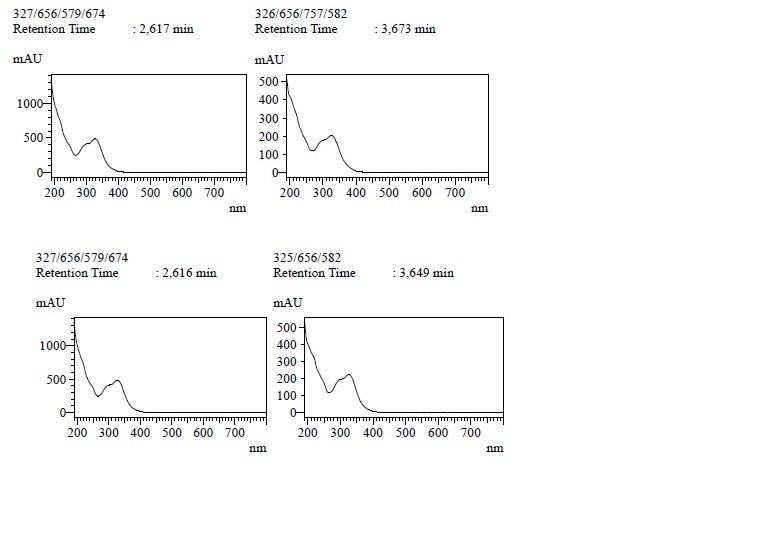


Analytical HPLC profile of *Vernonanthura nudiflora* extract at 10 mg/mL in ethanol. Mobile phase ACN/H_2_O 30:70. UV detection at 210 (A) and 280 (B) nm.

A


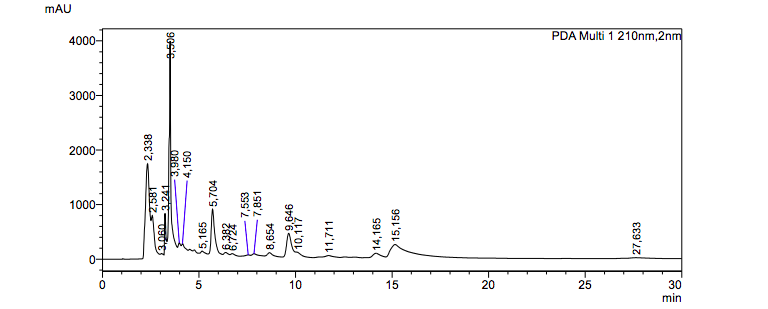


B


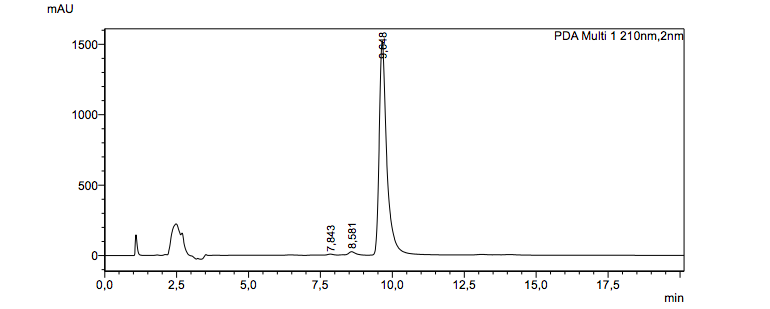


Analytical HPLC profile of *Gaillardia megapotamica* extract (A) and the isolated compound helenalin (red arrow) (B) at 10 mg/mL in methanol. Mobile phase ACN/H_2_O 30:70. UV detection at 210 nm.
